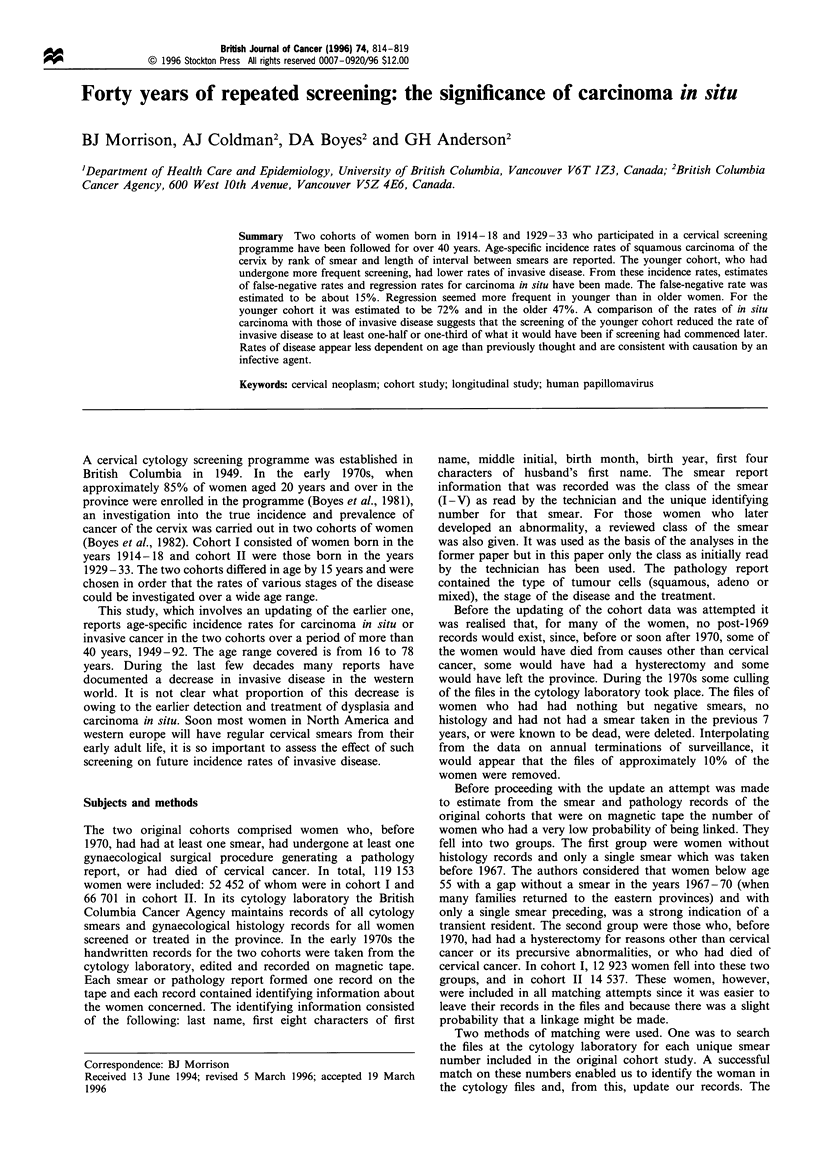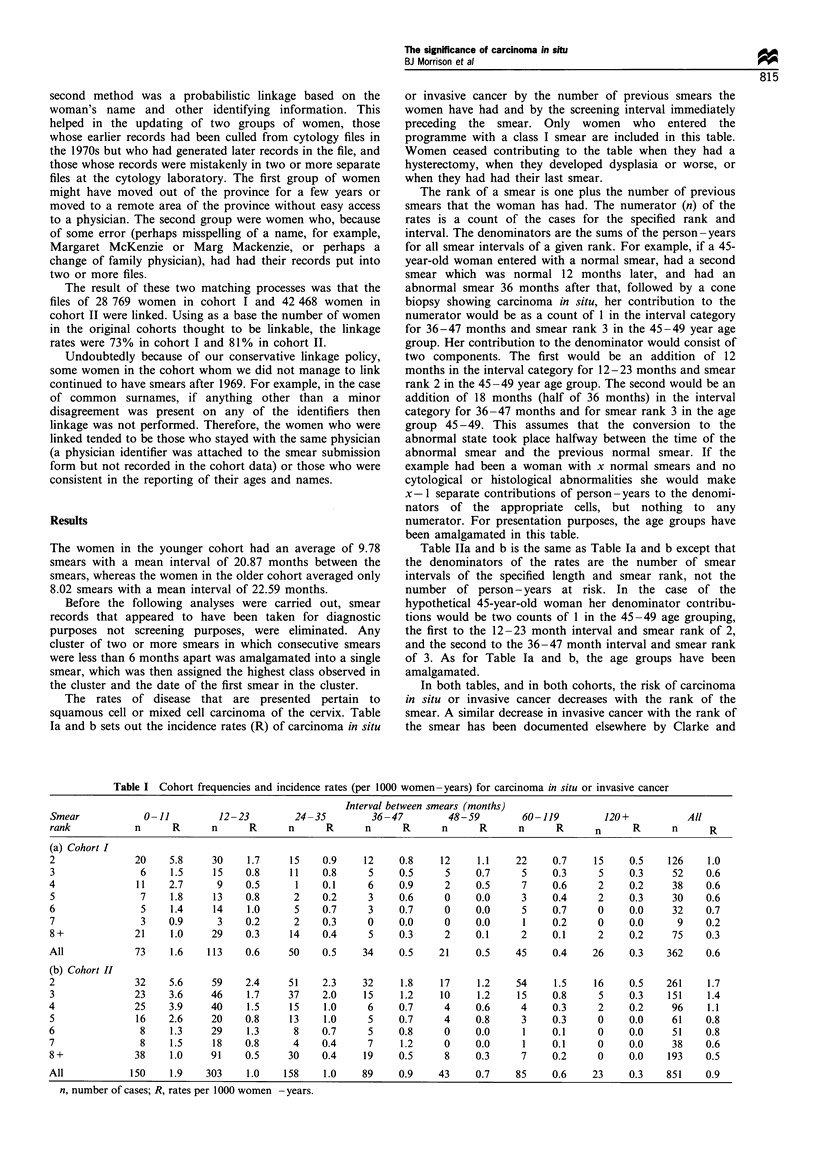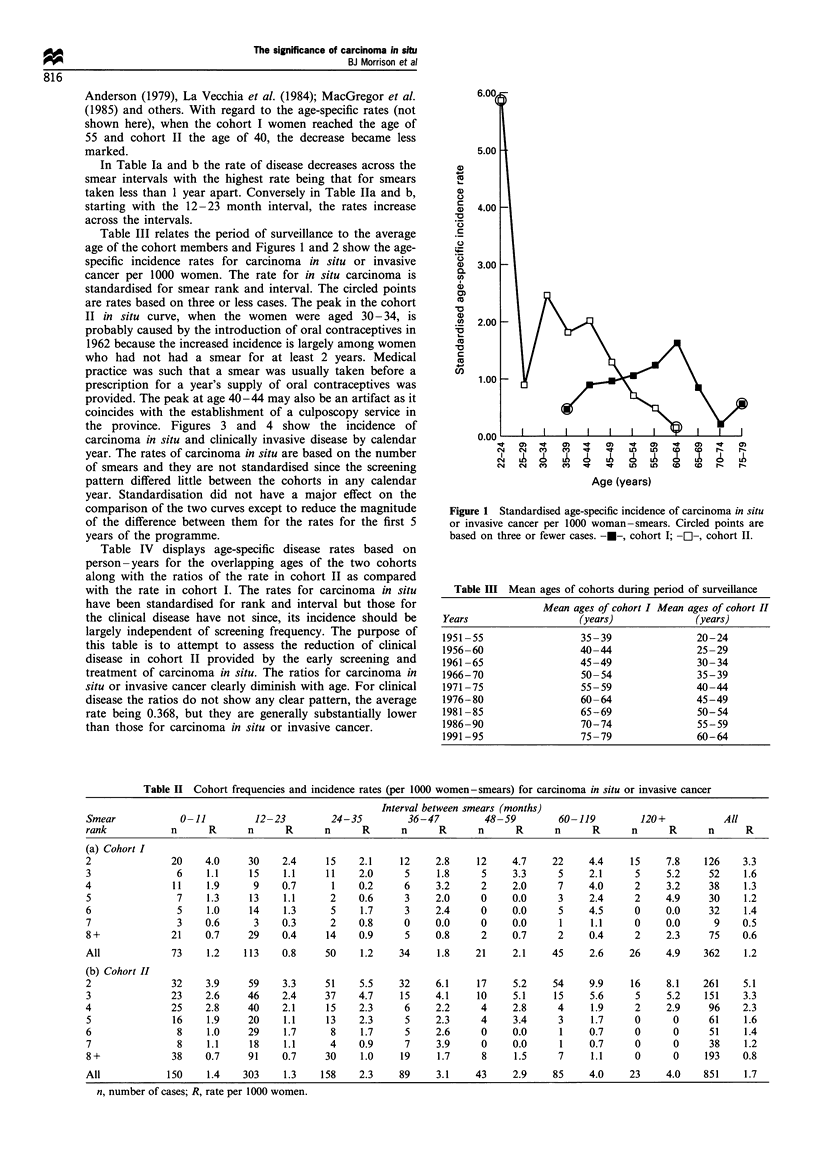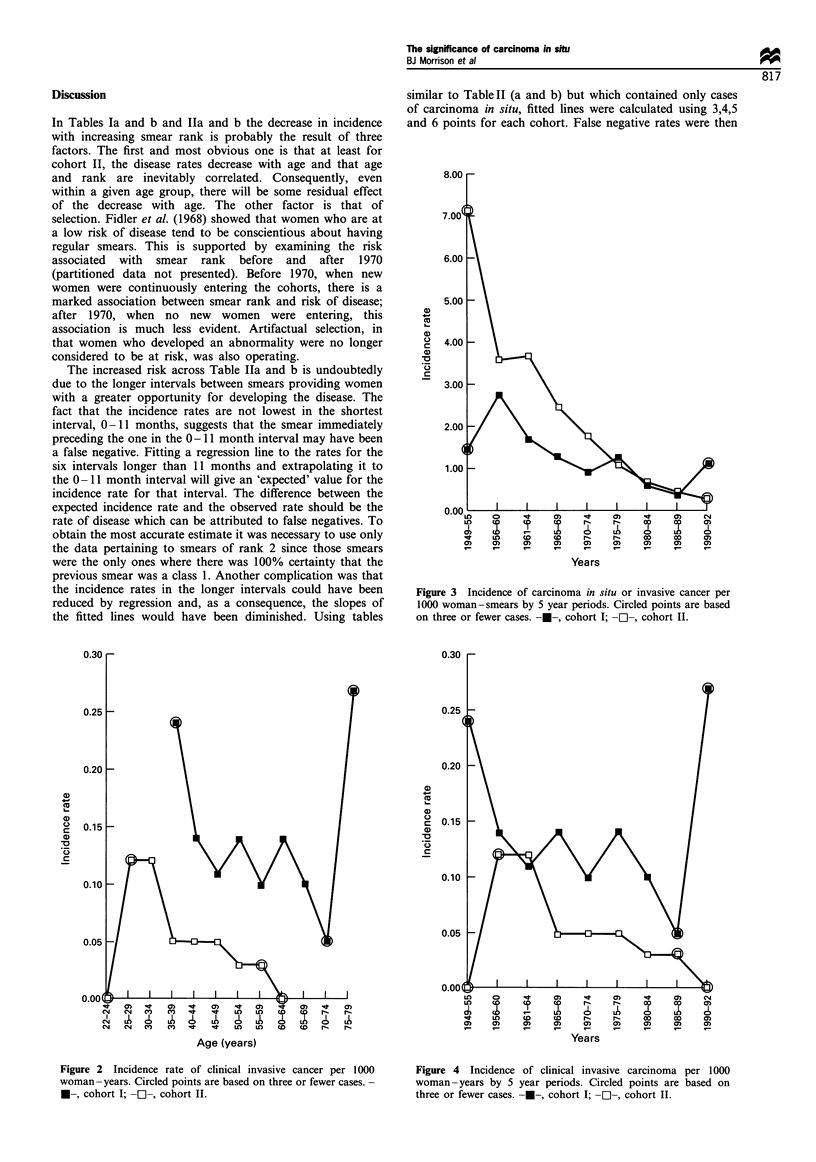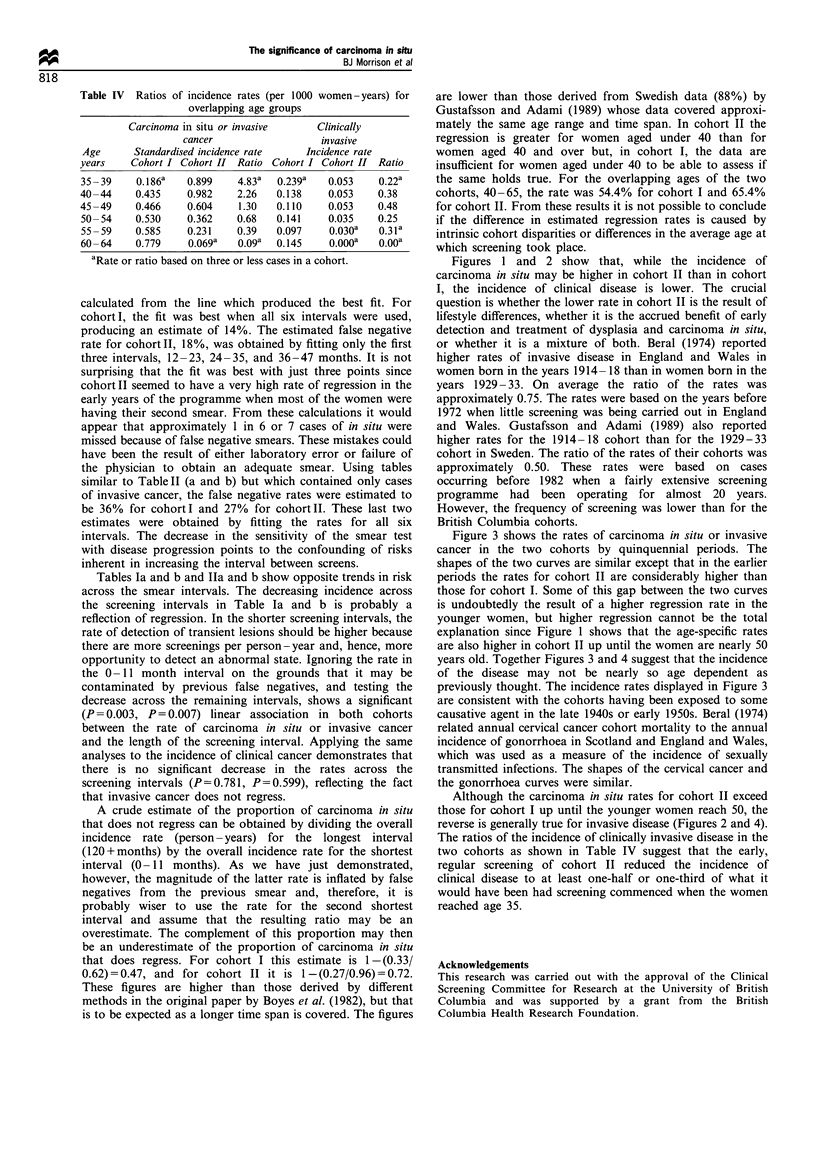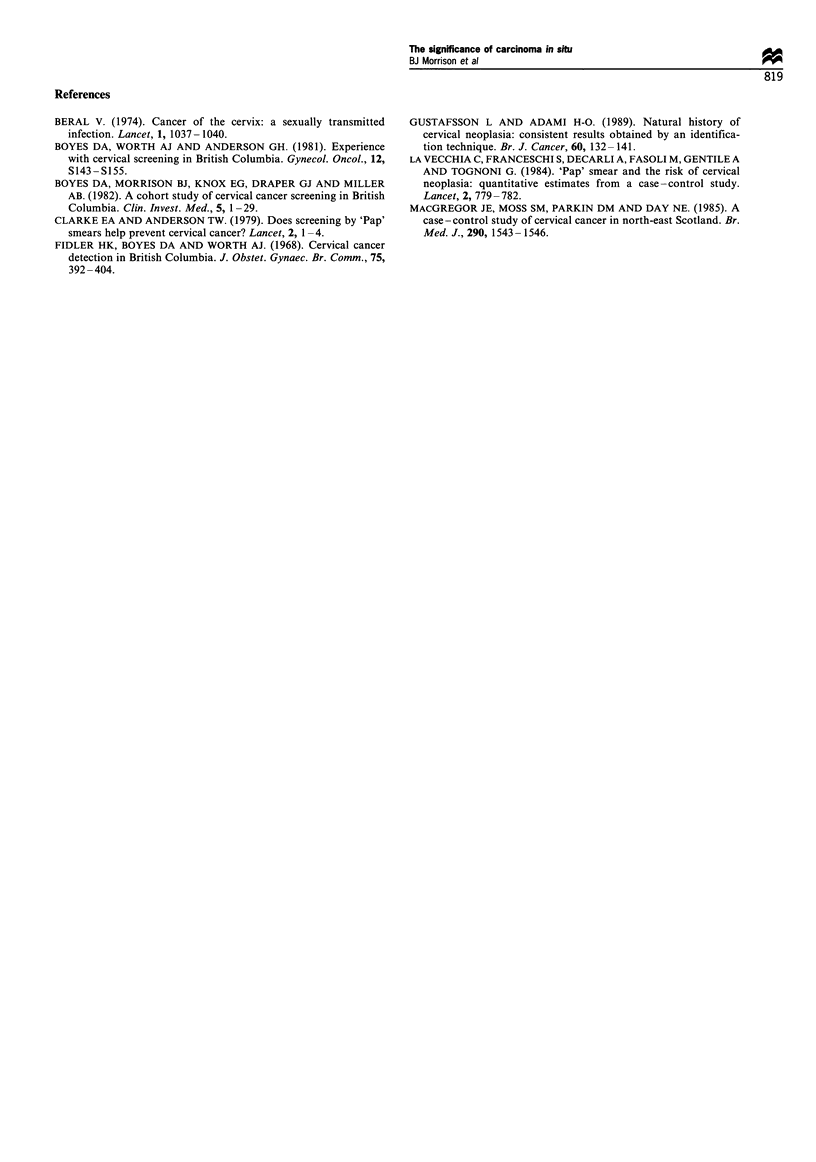# Forty years of repeated screening: the significance of carcinoma in situ.

**DOI:** 10.1038/bjc.1996.441

**Published:** 1996-09

**Authors:** B. J. Morrison, A. J. Coldman, D. A. Boyes, G. H. Anderson

**Affiliations:** Department of Health Care and Epidemiology, University of British Columbia, Vancouver, Canada.

## Abstract

Two cohorts of women born in 1914-18 and 1929-33 who participated in a cervical screening programme have been followed for over 40 years. Age-specific incidence rates of squamous carcinoma of the cervix by rank of smear and length of interval between smears are reported. The younger cohort, who had undergone more frequent screening, had lower rates of invasive disease. From these incidence rates, estimates of false-negative rates and regression rates for carcinoma in situ have been made. The false-negative rate was estimated to be about 15%. Regression seemed more frequent in younger than in older women. For the younger cohort it was estimated to be 72% and in the older 47%. A comparison of the rates of in situ carcinoma with those of invasive disease suggests that the screening of the younger cohort reduced the rate of invasive disease to at least one-half or one-third of what it would have been if screening had commenced later. Rates of disease appear less dependent on age than previously thought and are consistent with causation by an infective agent.


British Journal of Cancer (1996) 74, 814-819
© 1996 Stockton Press All rights reserved 0007-0920/96 $12.00

### Forty years of repeated screening: the significance of carcinoma *in situ*

BJ Morrison, AJ Coldman[2], DA Boyes[2] and GH Anderson[2]

[1]*Department of Health Care and Epidemiology, University of British Columbia, Vancouver V6T 1Z3, Canada;* [2]*British Columbia Cancer Agency, 600 West 10th Avenue, Vancouver V5Z 4E6, Canada.*

**Summary** Two cohorts of women born in 1914–18 and 1929–33 who participated in a cervical screening programme have been followed for over 40 years. Age-specific incidence rates of squamous carcinoma of the cervix by rank of smear and length of interval between smears are reported. The younger cohort, who had undergone more frequent screening, had lower rates of invasive disease. From these incidence rates, estimates of false-negative rates and regression rates for carcinoma *in situ* have been made. The false-negative rate was estimated to be about 15%. Regression seemed more frequent in younger than in older women. For the younger cohort it was estimated to be 72% and in the older 47%. A comparison of the rates of *in situ* carcinoma with those of invasive disease suggests that the screening of the younger cohort reduced the rate of invasive disease to at least one-half or one-third of what it would have been if screening had commenced later. Rates of disease appear less dependent on age than previously thought and are consistent with causation by an infective agent.

**Keywords:** cervical neoplasm; cohort study; longitudinal study; human papillomavirus

A cervical cytology screening programme was established in British Columbia in 1949. In the early 1970s, when approximately 85% of women aged 20 years and over in the province were enrolled in the programme (Boyes *et al.*, 1981), an investigation into the true incidence and prevalence of cancer of the cervix was carried out in two cohorts of women (Boyes *et al.*, 1982). Cohort I consisted of women born in the years 1914–18 and cohort II were those born in the years 1929–33. The two cohorts differed in age by 15 years and were chosen in order that the rates of various stages of the disease could be investigated over a wide age range.

This study, which involves an updating of the earlier one, reports age-specific incidence rates for carcinoma *in situ* or invasive cancer in the two cohorts over a period of more than 40 years, 1949–92. The age range covered is from 16 to 78 years. During the last few decades many reports have documented a decrease in invasive disease in the western world. It is not clear what proportion of this decrease is owing to the earlier detection and treatment of dysplasia and carcinoma *in situ*. Soon most women in North America and western europe will have regular cervical smears from their early adult life, it is so important to assess the effect of such screening on future incidence rates of invasive disease.

### Subjects and methods

The two original cohorts comprised women who, before 1970, had had at least one smear, had undergone at least one gynaecological surgical procedure generating a pathology report, or had died of cervical cancer. In total, 119 153 women were included: 52 452 of whom were in cohort I and 66 701 in cohort II. In its cytology laboratory the British Columbia Cancer Agency maintains records of all cytology smears and gynaecological histology records for all women screened or treated in the province. In the early 1970s the handwritten records for the two cohorts were taken from the cytology laboratory, edited and recorded on magnetic tape. Each smear or pathology report formed one record on the tape and each record contained identifying information about the women concerned. The identifying information consisted of the following: last name, first eight characters of first name, middle initial, birth month, birth year, first four characters of husband's first name. The smear report information that was recorded was the class of the smear (I–V) as read by the technician and the unique identifying number for that smear. For those women who later developed an abnormality, a reviewed class of the smear was also given. It was used as the basis of the analyses in the former paper but in this paper only the class as initially read by the technician has been used. The pathology report contained the type of tumour cells (squamous, adeno or mixed), the stage of the disease and the treatment.

Before the updating of the cohort data was attempted it was realised that, for many of the women, no post-1969 records would exist, since, before or soon after 1970, some of the women would have died from causes other than cervical cancer, some would have had a hysterectomy and some would have left the province. During the 1970s some culling of the files in the cytology laboratory took place. The files of women who had had nothing but negative smears, no histology and had not had a smear taken in the previous 7 years, or were known to be dead, were deleted. Interpolating from the data on annual terminations of surveillance, it would appear that the files of approximately 10% of the women were removed.

Before proceeding with the update an attempt was made to estimate from the smear and pathology records of the original cohorts that were on magnetic tape the number of women who had a very low probability of being linked. They fell into two groups. The first group were women without histology records and only a single smear which was taken before 1967. The authors considered that women below age 55 with a gap without a smear in the years 1967–70 (when many families returned to the eastern provinces) and with only a single smear preceding, was a strong indication of a transient resident. The second group were those who, before 1970, had had a hysterectomy for reasons other than cervical cancer or its precursive abnormalities, or who had died of cervical cancer. In cohort I, 12 923 women fell into these two groups, and in cohort II 14 537. These women, however, were included in all matching attempts since it was easier to leave their records in the files and because there was a slight probability that a linkage might be made.

Two methods of matching were used. One was to search the files at the cytology laboratory for each unique smear number included in the original cohort study. A successful match on these numbers enabled us to identify the woman in the cytology files and, from this, update our records. The

Correspondence: BJ Morrison
Received 13 June 1994; revised 5 March 1996; accepted 19 March 1996

second method was a probabilistic linkage based on the woman's name and other identifying information. This helped in the updating of two groups of women, those whose earlier records had been culled from cytology files in the 1970s but who had generated later records in the file, and those whose records were mistakenly in two or more separate files at the cytology laboratory. The first group of women might have moved out of the province for a few years or moved to a remote area of the province without easy access to a physician. The second group were women who, because of some error (perhaps misspelling of a name, for example, Margaret McKenzie or Marg Mackenzie, or perhaps a change of family physician), had had their records put into two or more files.

The result of these two matching processes was that the files of 28 769 women in cohort I and 42 468 women in cohort II were linked. Using as a base the number of women in the original cohorts thought to be linkable, the linkage rates were 73% in cohort I and 81% in cohort II.

Undoubtedly because of our conservative linkage policy, some women in the cohort whom we did not manage to link continued to have smears after 1969. For example, in the case of common surnames, if anything other than a minor disagreement was present on any of the identifiers then linkage was not performed. Therefore, the women who were linked tended to be those who stayed with the same physician (a physician identifier was attached to the smear submission form but not recorded in the cohort data) or those who were consistent in the reporting of their ages and names.

### Results

The women in the younger cohort had an average of 9.78 smears with a mean interval of 20.87 months between the smears, whereas the women in the older cohort averaged only 8.02 smears with a mean interval of 22.59 months.

Before the following analyses were carried out, smear records that appeared to have been taken for diagnostic purposes not screening purposes, were eliminated. Any cluster of two or more smears in which consecutive smears were less than 6 months apart was amalgamated into a single smear, which was then assigned the highest class observed in the cluster and the date of the first smear in the cluster.

The rates of disease that are presented pertain to squamous cell or mixed cell carcinoma of the cervix. Table Ia and b sets out the incidence rates (R) of carcinoma *in situ* or invasive cancer by the number of previous smears the women have had and by the screening interval immediately preceding the smear. Only women who entered the programme with a class I smear are included in this table. Women ceased contributing to the table when they had a hysterectomy, when they developed dysplasia or worse, or when they had had their last smear.

The rank of a smear is one plus the number of previous smears that the woman has had. The numerator (*n*) of the rates is a count of the cases for the specified rank and interval. The denominators are the sums of the person-years for all smear intervals of a given rank. For example, if a 45-year-old woman entered with a normal smear, had a second smear which was normal 12 months later, and had an abnormal smear 36 months after that, followed by a cone biopsy showing carcinoma *in situ*, her contribution to the numerator would be as a count of 1 in the interval category for 36-47 months and smear rank 3 in the 45-49 year age group. Her contribution to the denominator would consist of two components. The first would be an addition of 12 months in the interval category for 12-23 months and smear rank 2 in the 45-49 year age group. The second would be an addition of 18 months (half of 36 months) in the interval category for 36-47 months and for smear rank 3 in the age group 45-49. This assumes that the conversion to the abnormal state took place halfway between the time of the abnormal smear and the previous normal smear. If the example had been a woman with $x$ normal smears and no cytological or histological abnormalities she would make $x-1$ separate contributions of person-years to the denominators of the appropriate cells, but nothing to any numerator. For presentation purposes, the age groups have been amalgamated in this table.

Table IIa and b is the same as Table Ia and b except that the denominators of the rates are the number of smear intervals of the specified length and smear rank, not the number of person-years at risk. In the case of the hypothetical 45-year-old woman her denominator contributions would be two counts of 1 in the 45-49 age grouping, the first to the 12-23 month interval and smear rank of 2, and the second to the 36-47 month interval and smear rank of 3. As for Table Ia and b, the age groups have been amalgamated.

In both tables, and in both cohorts, the risk of carcinoma *in situ* or invasive cancer decreases with the rank of the smear. A similar decrease in invasive cancer with the rank of the smear has been documented elsewhere by Clarke and

**Table I** Cohort frequencies and incidence rates (per 1000 women-years) for carcinoma *in situ* or invasive cancer

| *Smear rank* | *0-11* n | *0-11* R | *12-23* n | *12-23* R | *24-35* n | *24-35* R | *36-47* n | *36-47* R | *48-59* n | *48-59* R | *60-119* n | *60-119* R | *120+* n | *120+* R | *All* n | *All* R |
|---|---|---|---|---|---|---|---|---|---|---|---|---|---|---|---|---|
| (a) *Cohort I* | | | | | | | | | | | | | | | | |
| 2 | 20 | 5.8 | 30 | 1.7 | 15 | 0.9 | 12 | 0.8 | 12 | 1.1 | 22 | 0.7 | 15 | 0.5 | 126 | 1.0 |
| 3 | 6 | 1.5 | 15 | 0.8 | 11 | 0.8 | 5 | 0.5 | 5 | 0.7 | 5 | 0.3 | 5 | 0.3 | 52 | 0.6 |
| 4 | 11 | 2.7 | 9 | 0.5 | 1 | 0.1 | 6 | 0.9 | 2 | 0.5 | 7 | 0.6 | 2 | 0.2 | 38 | 0.6 |
| 5 | 7 | 1.8 | 13 | 0.8 | 2 | 0.2 | 3 | 0.6 | 0 | 0.0 | 3 | 0.4 | 2 | 0.3 | 30 | 0.6 |
| 6 | 5 | 1.4 | 14 | 1.0 | 5 | 0.7 | 3 | 0.7 | 0 | 0.0 | 5 | 0.7 | 0 | 0.0 | 32 | 0.7 |
| 7 | 3 | 0.9 | 3 | 0.2 | 2 | 0.3 | 0 | 0.0 | 0 | 0.0 | 1 | 0.2 | 0 | 0.0 | 9 | 0.2 |
| 8+ | 21 | 1.0 | 29 | 0.3 | 14 | 0.4 | 5 | 0.3 | 2 | 0.1 | 2 | 0.1 | 2 | 0.2 | 75 | 0.3 |
| All | 73 | 1.6 | 113 | 0.6 | 50 | 0.5 | 34 | 0.5 | 21 | 0.5 | 45 | 0.4 | 26 | 0.3 | 362 | 0.6 |
| (b) *Cohort II* | | | | | | | | | | | | | | | | |
| 2 | 32 | 5.6 | 59 | 2.4 | 51 | 2.3 | 32 | 1.8 | 17 | 1.2 | 54 | 1.5 | 16 | 0.5 | 261 | 1.7 |
| 3 | 23 | 3.6 | 46 | 1.7 | 37 | 2.0 | 15 | 1.2 | 10 | 1.2 | 15 | 0.8 | 5 | 0.3 | 151 | 1.4 |
| 4 | 25 | 3.9 | 40 | 1.5 | 15 | 1.0 | 6 | 0.7 | 4 | 0.6 | 4 | 0.3 | 2 | 0.2 | 96 | 1.1 |
| 5 | 16 | 2.6 | 20 | 0.8 | 13 | 1.0 | 5 | 0.7 | 4 | 0.8 | 3 | 0.3 | 0 | 0.0 | 61 | 0.8 |
| 6 | 8 | 1.3 | 29 | 1.3 | 8 | 0.7 | 5 | 0.8 | 0 | 0.0 | 1 | 0.1 | 0 | 0.0 | 51 | 0.8 |
| 7 | 8 | 1.5 | 18 | 0.8 | 4 | 0.4 | 7 | 1.2 | 0 | 0.0 | 1 | 0.1 | 0 | 0.0 | 38 | 0.6 |
| 8+ | 38 | 1.0 | 91 | 0.5 | 30 | 0.4 | 19 | 0.5 | 8 | 0.3 | 7 | 0.2 | 0 | 0.0 | 193 | 0.5 |
| All | 150 | 1.9 | 303 | 1.0 | 158 | 1.0 | 89 | 0.9 | 43 | 0.7 | 85 | 0.6 | 23 | 0.3 | 851 | 0.9 |

Column groups: *Interval between smears (months)*.

*n*, number of cases; *R*, rates per 1000 women -years.

Anderson (1979), La Vecchia *et al.* (1984); MacGregor *et al.* (1985) and others. With regard to the age-specific rates (not shown here), when the cohort I women reached the age of 55 and cohort II the age of 40, the decrease became less marked.

In Table Ia and b the rate of disease decreases across the smear intervals with the highest rate being that for smears taken less than 1 year apart. Conversely in Table IIa and b, starting with the 12–23 month interval, the rates increase across the intervals.

Table III relates the period of surveillance to the average age of the cohort members and Figures 1 and 2 show the age-specific incidence rates for carcinoma *in situ* or invasive cancer per 1000 women. The rate for *in situ* carcinoma is standardised for smear rank and interval. The circled points are rates based on three or less cases. The peak in the cohort II *in situ* curve, when the women were aged 30–34, is probably caused by the introduction of oral contraceptives in 1962 because the increased incidence is largely among women who had not had a smear for at least 2 years. Medical practice was such that a smear was usually taken before a prescription for a year's supply of oral contraceptives was provided. The peak at age 40–44 may also be an artifact as it coincides with the establishment of a culposcopy service in the province. Figures 3 and 4 show the incidence of carcinoma *in situ* and clinically invasive disease by calendar year. The rates of carcinoma *in situ* are based on the number of smears and they are not standardised since the screening pattern differed little between the cohorts in any calendar year. Standardisation did not have a major effect on the comparison of the two curves except to reduce the magnitude of the difference between them for the rates for the first 5 years of the programme.

Table IV displays age-specific disease rates based on person–years for the overlapping ages of the two cohorts along with the ratios of the rate in cohort II as compared with the rate in cohort I. The rates for carcinoma *in situ* have been standardised for rank and interval but those for the clinical disease have not since, its incidence should be largely independent of screening frequency. The purpose of this table is to attempt to assess the reduction of clinical disease in cohort II provided by the early screening and treatment of carcinoma *in situ*. The ratios for carcinoma *in situ* or invasive cancer clearly diminish with age. For clinical disease the ratios do not show any clear pattern, the average rate being 0.368, but they are generally substantially lower than those for carcinoma *in situ* or invasive cancer.

**Figure 1** Standardised age-specific incidence of carcinoma *in situ* or invasive cancer per 1000 woman–smears. Circled points are based on three or fewer cases. –■–, cohort I; –□–, cohort II.

**Table III** Mean ages of cohorts during period of surveillance

| *Years* | *Mean ages of cohort I (years)* | *Mean ages of cohort II (years)* |
|---|---|---|
| 1951–55 | 35–39 | 20–24 |
| 1956–60 | 40–44 | 25–29 |
| 1961–65 | 45–49 | 30–34 |
| 1966–70 | 50–54 | 35–39 |
| 1971–75 | 55–59 | 40–44 |
| 1976–80 | 60–64 | 45–49 |
| 1981–85 | 65–69 | 50–54 |
| 1986–90 | 70–74 | 55–59 |
| 1991–95 | 75–79 | 60–64 |

**Table II** Cohort frequencies and incidence rates (per 1000 women–smears) for carcinoma *in situ* or invasive cancer

| *Smear rank* | *0–11* n | R | *12–23* n | R | *24–35* n | R | *36–47* n | R | *48–59* n | R | *60–119* n | R | *120+* n | R | *All* n | R |
|---|---|---|---|---|---|---|---|---|---|---|---|---|---|---|---|---|
| (a) *Cohort I* | | | | | | | | | | | | | | | | |
| 2 | 20 | 4.0 | 30 | 2.4 | 15 | 2.1 | 12 | 2.8 | 12 | 4.7 | 22 | 4.4 | 15 | 7.8 | 126 | 3.3 |
| 3 | 6 | 1.1 | 15 | 1.1 | 11 | 2.0 | 5 | 1.8 | 5 | 3.3 | 5 | 2.1 | 5 | 5.2 | 52 | 1.6 |
| 4 | 11 | 1.9 | 9 | 0.7 | 1 | 0.2 | 6 | 3.2 | 2 | 2.0 | 7 | 4.0 | 2 | 3.2 | 38 | 1.3 |
| 5 | 7 | 1.3 | 13 | 1.1 | 2 | 0.6 | 3 | 2.0 | 0 | 0.0 | 3 | 2.4 | 2 | 4.9 | 30 | 1.2 |
| 6 | 5 | 1.0 | 14 | 1.3 | 5 | 1.7 | 3 | 2.4 | 0 | 0.0 | 5 | 4.5 | 0 | 0.0 | 32 | 1.4 |
| 7 | 3 | 0.6 | 3 | 0.3 | 2 | 0.8 | 0 | 0.0 | 0 | 0.0 | 1 | 1.1 | 0 | 0.0 | 9 | 0.5 |
| 8+ | 21 | 0.7 | 29 | 0.4 | 14 | 0.9 | 5 | 0.8 | 2 | 0.7 | 2 | 0.4 | 2 | 2.3 | 75 | 0.6 |
| All | 73 | 1.2 | 113 | 0.8 | 50 | 1.2 | 34 | 1.8 | 21 | 2.1 | 45 | 2.6 | 26 | 4.9 | 362 | 1.2 |
| (b) *Cohort II* | | | | | | | | | | | | | | | | |
| 2 | 32 | 3.9 | 59 | 3.3 | 51 | 5.5 | 32 | 6.1 | 17 | 5.2 | 54 | 9.9 | 16 | 8.1 | 261 | 5.1 |
| 3 | 23 | 2.6 | 46 | 2.4 | 37 | 4.7 | 15 | 4.1 | 10 | 5.1 | 15 | 5.6 | 5 | 5.2 | 151 | 3.3 |
| 4 | 25 | 2.8 | 40 | 2.1 | 15 | 2.3 | 6 | 2.2 | 4 | 2.8 | 4 | 1.9 | 2 | 2.9 | 96 | 2.3 |
| 5 | 16 | 1.9 | 20 | 1.1 | 13 | 2.3 | 5 | 2.3 | 4 | 3.4 | 3 | 1.7 | 0 | 0 | 61 | 1.6 |
| 6 | 8 | 1.0 | 29 | 1.7 | 8 | 1.7 | 5 | 2.6 | 0 | 0.0 | 1 | 0.7 | 0 | 0 | 51 | 1.4 |
| 7 | 8 | 1.1 | 18 | 1.1 | 4 | 0.9 | 7 | 3.9 | 0 | 0.0 | 1 | 0.7 | 0 | 0 | 38 | 1.2 |
| 8+ | 38 | 0.7 | 91 | 0.7 | 30 | 1.0 | 19 | 1.7 | 8 | 1.5 | 7 | 1.1 | 0 | 0 | 193 | 0.8 |
| All | 150 | 1.4 | 303 | 1.3 | 158 | 2.3 | 89 | 3.1 | 43 | 2.9 | 85 | 4.0 | 23 | 4.0 | 851 | 1.7 |

Interval between smears (months)

*n*, number of cases; *R*, rate per 1000 women.

### Discussion

In Tables Ia and b and IIa and b the decrease in incidence with increasing smear rank is probably the result of three factors. The first and most obvious one is that at least for cohort II, the disease rates decrease with age and that age and rank are inevitably correlated. Consequently, even within a given age group, there will be some residual effect of the decrease with age. The other factor is that of selection. Fidler *et al.* (1968) showed that women who are at a low risk of disease tend to be conscientious about having regular smears. This is supported by examining the risk associated with smear rank before and after 1970 (partitioned data not presented). Before 1970, when new women were continuously entering the cohorts, there is a marked association between smear rank and risk of disease; after 1970, when no new women were entering, this association is much less evident. Artifactual selection, in that women who developed an abnormality were no longer considered to be at risk, was also operating.

The increased risk across Table IIa and b is undoubtedly due to the longer intervals between smears providing women with a greater opportunity for developing the disease. The fact that the incidence rates are not lowest in the shortest interval, 0–11 months, suggests that the smear immediately preceding the one in the 0–11 month interval may have been a false negative. Fitting a regression line to the rates for the six intervals longer than 11 months and extrapolating it to the 0–11 month interval will give an 'expected' value for the incidence rate for that interval. The difference between the expected incidence rate and the observed rate should be the rate of disease which can be attributed to false negatives. To obtain the most accurate estimate it was necessary to use only the data pertaining to smears of rank 2 since those smears were the only ones where there was 100% certainty that the previous smear was a class 1. Another complication was that the incidence rates in the longer intervals could have been reduced by regression and, as a consequence, the slopes of the fitted lines would have been diminished. Using tables similar to Table II (a and b) but which contained only cases of carcinoma *in situ*, fitted lines were calculated using 3,4,5 and 6 points for each cohort. False negative rates were then

**Figure 3** Incidence of carcinoma *in situ* or invasive cancer per 1000 woman–smears by 5 year periods. Circled points are based on three or fewer cases. –■–, cohort I; –□–, cohort II.

**Figure 2** Incidence rate of clinical invasive cancer per 1000 woman–years. Circled points are based on three or fewer cases. –■–, cohort I; –□–, cohort II.

**Figure 4** Incidence of clinical invasive carcinoma per 1000 woman–years by 5 year periods. Circled points are based on three or fewer cases. –■–, cohort I; –□–, cohort II.

**Table IV** Ratios of incidence rates (per 1000 women-years) for overlapping age groups

| Age years | *Carcinoma* in situ *or invasive cancer* Standardised incidence rate Cohort I | Cohort II | Ratio | *Clinically invasive* Incidence rate Cohort I | Cohort II | Ratio |
|---|---|---|---|---|---|---|
| 35-39 | 0.186[a] | 0.899 | 4.83[a] | 0.239[a] | 0.053 | 0.22[a] |
| 40-44 | 0.435 | 0.982 | 2.26 | 0.138 | 0.053 | 0.38 |
| 45-49 | 0.466 | 0.604 | 1.30 | 0.110 | 0.053 | 0.48 |
| 50-54 | 0.530 | 0.362 | 0.68 | 0.141 | 0.035 | 0.25 |
| 55-59 | 0.585 | 0.231 | 0.39 | 0.097 | 0.030[a] | 0.31[a] |
| 60-64 | 0.779 | 0.069[a] | 0.09[a] | 0.145 | 0.000[a] | 0.00[a] |

[a]Rate or ratio based on three or less cases in a cohort.

calculated from the line which produced the best fit. For cohort I, the fit was best when all six intervals were used, producing an estimate of 14%. The estimated false negative rate for cohort II, 18%, was obtained by fitting only the first three intervals, 12-23, 24-35, and 36-47 months. It is not surprising that the fit was best with just three points since cohort II seemed to have a very high rate of regression in the early years of the programme when most of the women were having their second smear. From these calculations it would appear that approximately 1 in 6 or 7 cases of *in situ* were missed because of false negative smears. These mistakes could have been the result of either laboratory error or failure of the physician to obtain an adequate smear. Using tables similar to Table II (a and b) but which contained only cases of invasive cancer, the false negative rates were estimated to be 36% for cohort I and 27% for cohort II. These last two estimates were obtained by fitting the rates for all six intervals. The decrease in the sensitivity of the smear test with disease progression points to the confounding of risks inherent in increasing the interval between screens.

Tables Ia and b and IIa and b show opposite trends in risk across the smear intervals. The decreasing incidence across the screening intervals in Table Ia and b is probably a reflection of regression. In the shorter screening intervals, the rate of detection of transient lesions should be higher because there are more screenings per person-year and, hence, more opportunity to detect an abnormal state. Ignoring the rate in the 0-11 month interval on the grounds that it may be contaminated by previous false negatives, and testing the decrease across the remaining intervals, shows a significant ($P=0.003$, $P=0.007$) linear association in both cohorts between the rate of carcinoma *in situ* or invasive cancer and the length of the screening interval. Applying the same analyses to the incidence of clinical cancer demonstrates that there is no significant decrease in the rates across the screening intervals ($P=0.781$, $P=0.599$), reflecting the fact that invasive cancer does not regress.

A crude estimate of the proportion of carcinoma *in situ* that does not regress can be obtained by dividing the overall incidence rate (person-years) for the longest interval (120+months) by the overall incidence rate for the shortest interval (0-11 months). As we have just demonstrated, however, the magnitude of the latter rate is inflated by false negatives from the previous smear and, therefore, it is probably wiser to use the rate for the second shortest interval and assume that the resulting ratio may be an overestimate. The complement of this proportion may then be an underestimate of the proportion of carcinoma *in situ* that does regress. For cohort I this estimate is $1-(0.33/0.62)=0.47$, and for cohort II it is $1-(0.27/0.96)=0.72$. These figures are higher than those derived by different methods in the original paper by Boyes *et al.* (1982), but that is to be expected as a longer time span is covered. The figures are lower than those derived from Swedish data (88%) by Gustafsson and Adami (1989) whose data covered approximately the same age range and time span. In cohort II the regression is greater for women aged under 40 than for women aged 40 and over but, in cohort I, the data are insufficient for women aged under 40 to be able to assess if the same holds true. For the overlapping ages of the two cohorts, 40-65, the rate was 54.4% for cohort I and 65.4% for cohort II. From these results it is not possible to conclude if the difference in estimated regression rates is caused by intrinsic cohort disparities or differences in the average age at which screening took place.

Figures 1 and 2 show that, while the incidence of carcinoma *in situ* may be higher in cohort II than in cohort I, the incidence of clinical disease is lower. The crucial question is whether the lower rate in cohort II is the result of lifestyle differences, whether it is the accrued benefit of early detection and treatment of dysplasia and carcinoma *in situ*, or whether it is a mixture of both. Beral (1974) reported higher rates of invasive disease in England and Wales in women born in the years 1914-18 than in women born in the years 1929-33. On average the ratio of the rates was approximately 0.75. The rates were based on the years before 1972 when little screening was being carried out in England and Wales. Gustafsson and Adami (1989) also reported higher rates for the 1914-18 cohort than for the 1929-33 cohort in Sweden. The ratio of the rates of their cohorts was approximately 0.50. These rates were based on cases occurring before 1982 when a fairly extensive screening programme had been operating for almost 20 years. However, the frequency of screening was lower than for the British Columbia cohorts.

Figure 3 shows the rates of carcinoma *in situ* or invasive cancer in the two cohorts by quinquennial periods. The shapes of the two curves are similar except that in the earlier periods the rates for cohort II are considerably higher than those for cohort I. Some of this gap between the two curves is undoubtedly the result of a higher regression rate in the younger women, but higher regression cannot be the total explanation since Figure 1 shows that the age-specific rates are also higher in cohort II up until the women are nearly 50 years old. Together Figures 3 and 4 suggest that the incidence of the disease may not be nearly so age dependent as previously thought. The incidence rates displayed in Figure 3 are consistent with the cohorts having been exposed to some causative agent in the late 1940s or early 1950s. Beral (1974) related annual cervical cancer cohort mortality to the annual incidence of gonorrhoea in Scotland and England and Wales, which was used as a measure of the incidence of sexually transmitted infections. The shapes of the cervical cancer and the gonorrhoea curves were similar.

Although the carcinoma *in situ* rates for cohort II exceed those for cohort I up until the younger women reach 50, the reverse is generally true for invasive disease (Figures 2 and 4). The ratios of the incidence of clinically invasive disease in the two cohorts as shown in Table IV suggest that the early, regular screening of cohort II reduced the incidence of clinical disease to at least one-half or one-third of what it would have been had screening commenced when the women reached age 35.

**Acknowledgements**
This research was carried out with the approval of the Clinical Screening Committee for Research at the University of British Columbia and was supported by a grant from the British Columbia Health Research Foundation.

### References

BERAL V. (1974). Cancer of the cervix: a sexually transmitted infection. *Lancet*, **1**, 1037–1040.

BOYES DA, WORTH AJ AND ANDERSON GH. (1981). Experience with cervical screening in British Columbia. *Gynecol. Oncol.*, **12**, S143–S155.

BOYES DA, MORRISON BJ, KNOX EG, DRAPER GJ AND MILLER AB. (1982). A cohort study of cervical cancer screening in British Columbia. *Clin. Invest. Med.*, **5**, 1–29.

CLARKE EA AND ANDERSON TW. (1979). Does screening by 'Pap' smears help prevent cervical cancer? *Lancet*, **2**, 1–4.

FIDLER HK, BOYES DA AND WORTH AJ. (1968). Cervical cancer detection in British Columbia. *J. Obstet. Gynaec. Br. Comm.*, **75**, 392–404.

GUSTAFSSON L AND ADAMI H-O. (1989). Natural history of cervical neoplasia: consistent results obtained by an identification technique. *Br. J. Cancer*, **60**, 132–141.

LA VECCHIA C, FRANCESCHI S, DECARLI A, FASOLI M, GENTILE A AND TOGNONI G. (1984). 'Pap' smear and the risk of cervical neoplasia: quantitative estimates from a case–control study. *Lancet*, **2**, 779–782.

MACGREGOR JE, MOSS SM, PARKIN DM AND DAY NE. (1985). A case–control study of cervical cancer in north-east Scotland. *Br. Med. J.*, **290**, 1543–1546.